# Supplementary material for: Surface Motility Favors Codependent Interaction between Pseudomonas aeruginosa and Burkholderia cenocepacia
Source: mSphere. 2022 Jul 7;7(4):e00153-22. doi: 10.1128/msphere.00153-22 (PMC9429929; doi:10.1128/msphere.00153-22)
Supplement: Figure S1 [file msphere.00153-22-sf001.pdf]

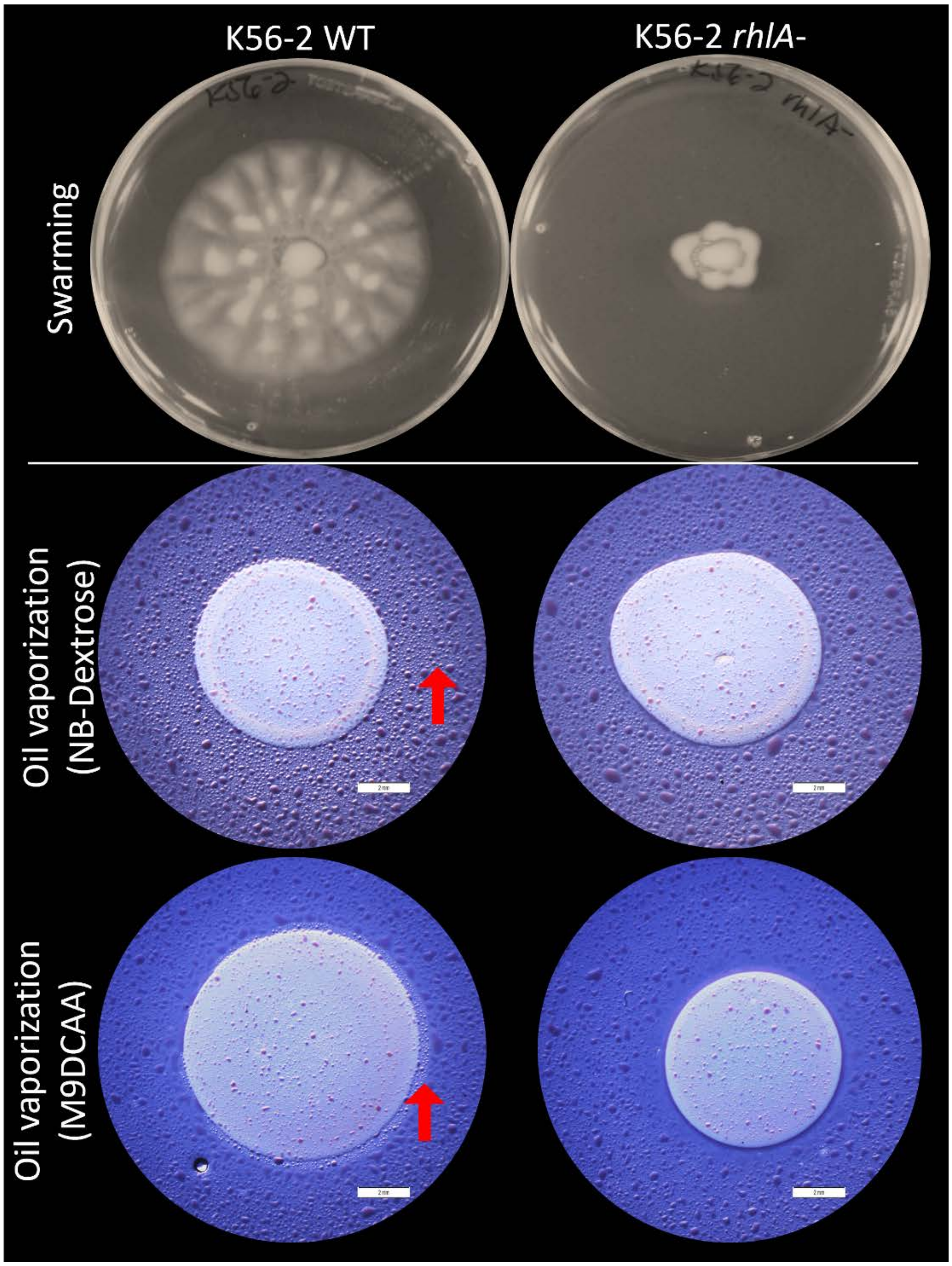

Figure S1. ***Burkholderia cenocepacia*** **K56-2 can swarm and produces an unidentified surface-active agent.** **TOP** Swarming of wildtype K56-2 and *rhlA*- mutant when grown overnight on semi-solid NB supplemented with dextrose and 0.5% agar. **BOTTOM** Oil vaporization after overnight growth on NB-Dextrose with 1.5% agar or M9DCAA medium with 1,5% agar reveals a light zone of diffusible surface-active molecules released by K56-2 WT (red arrow), absent from the *rhlA*- mutant. Sudan IV-colored mineral oil is vaporized onto the grown colonies. White scale bar is 2 mm.
